# Microbial community variations in human salivary samples with different body mass index for forensic research: a pilot study

**DOI:** 10.3389/fmicb.2026.1783246

**Published:** 2026-04-21

**Authors:** Jingyan Ji, Shunyi Tian, Han Zhang, Meiqing Yang, Zhengyang Song, Qian Zhang, Xiaoye Jin, Jiang Huang

**Affiliations:** 1 Department of Forensic Medicine, Guizhou Medical University, Guiyang, China; 2 Department of Basic Medical Sciences, Guizhou Medical University, Guiyang, China; 3 Institute of Forensic Science, Fudan University, Shanghai, China; 4 School of Public Health, Key Laboratory of Environmental Pollution Monitoring and Disease Control, Ministry of Education, Guizhou Medical University, Guiyang, China; 5 Toxicology Testing Center of Guizhou Medical University, Guiyang, China

**Keywords:** 16S rRNA, body mass index, forensic science, microbial community, salivary

## Abstract

Body mass index (BMI) is a crucial phenotypic feature with significant application value in forensic investigations; however, inferring BMI from forensic-related biological samples such as saliva remains challenging. In this study, saliva samples were collected and subjected to 16S rRNA sequencing to characterize microbial community composition, and BMI-associated microbial markers were screened using linear discriminant analysis effect size and the Kruskal–Wallis rank-sum test. A random forest model was subsequently constructed to infer BMI categories based on the selected microbial markers. The results showed that twenty-two microbial taxa, including the genera Neisseria, Veillonella, Prevotella, Streptococcus, and Achromobacter, exhibited significantly different abundance distributions among BMI groups and could serve as BMI-associated microbial indicators. Principal coordinates analysis demonstrated a clear separation between normal-weight and overweight groups, and the random forest model accurately inferred BMI categories for most samples. These findings indicate that saliva-associated microbial markers have potential as valuable indicators for BMI inference, providing a promising tool for forensic research.

## 1 Introduction

Forensic medicine plays a crucial role in the judicial trial system by providing scientific methods and evidence for the resolution of criminal cases. Over time, DNA profiling has become an increasingly important tool in forensic casework, enabling the identification of individuals involved in criminal cases; this is often referred to as forensic DNA fingerprinting (Jeffreys, 2005; Jobling, 2013; Romano et al., 2019). Conventional forensic DNA fingerprinting testing primarily involves extracting DNA profile information from biological materials (such as hair, blood, semen, and saliva) found at crime scenes. This information is then compared with the forensic DNA database or the reference DNA profile of a suspect to determine the identity of the perpetrator and other relevant facts of the crime. However, due to environmental factors and long-term exposure *in vitro*, the DNA in collected crime scene samples is prone to degradation and is often available only in trace amounts. Moreover, the DNA profile obtained from the evidence may not match the suspect's reference DNA profile or the forensic database. As a result, the information obtained through forensic DNA fingerprinting methods may not always be available (Dash and Das, 2017; Haddrill, 2021). In cases where other evidence is lacking, investigators frequently rely on eyewitness testimony to gather information. However, this type of evidence can be highly subjective, which means that it is not always reliable (Wells et al., 2006). In situations where standard DNA profiles and eyewitness accounts are unavailable, researchers have explored the use of externally visible characteristics (e.g., gender, age, hair color, weight, and eye color) obtained from DNA evidence profiles. This approach has proven useful in assisting investigators in narrowing down the investigation scope and is known as forensic DNA phenotyping, which involves establishing the connection between DNA profiles and the outward appearance of individuals (Chaitanya et al., 2018; Dabas et al., 2022; Kayser, 2015; Kayser et al., 2023; Kayser and Schneider, 2009; Schneider et al., 2019). For instance, (Chaitanya et al. 2018) developed the HIrisPlex-S DNA detection system consisting of 41 phenotype-related single-nucleotide polymorphisms, which could predict the eye, hair, and skin colors of unknown individuals. These externally visible characteristics are vital components in forensic DNA phenotyping. Specifically, when viewing video surveillance footage or eyewitness descriptions of suspects, the body size is often a significant factor considered by forensic investigators. Thus, the study of body size could also hold great value for forensic research. Body mass index (BMI) is commonly used as an alternative method to assess body size. However, due to incomplete DNA profiles from low DNA content and poor-quality biological samples found at crime scenes, it is difficult for forensic researchers to infer BMI of unknown individuals by assessing DNA markers (Cho et al., 2021; Lee et al., 2022). This results in the low practical application of BMI inference in forensic studies. To address these limitations, it's necessary to develop a more reliable and effective approach to inferring individual's BMI.

Forensic microbiology, which explores the population characteristics of microorganisms, has emerged as a new research direction for forensic scientists, particularly for trace or degraded samples (Metcalf et al., 2017). Microbes are ubiquitous, and it has been found that the total number of microbial communities in the human body is significantly higher than the number of human cells, ranging from 10 to 100 trillion microbiomes (Costello et al., 2009; Li et al., 2022). Their abundance, diversity, and resilience make them highly valuable and promising in various fields, especially in forensic science. Notably, microbial DNA, being circular and membrane-bound, is less prone to degradation compared to human DNA. Consequently, even trace samples collected from crime scenes may contain a rich assortment of microorganisms that can provide valuable information for forensic analyses. Thus, microbial DNA overcomes the disadvantages of trace and easy degradation of human DNA at the crime scene, thus showing great potential for forensic research. Previous studies have successfully established a correlation between BMI and the composition of intestinal microorganisms (Lv et al., 2019; Palmas et al., 2021; Yun et al., 2017). However, feces are not common samples in forensic scenes, which makes them provide limited application value in forensic applications. Conversely, saliva is a commonly encountered biological sample in the majority of crime scenes and can offer a more feasible option for analysis in forensic contexts (Smith and Giulivi, 2024). The human oral microbiome ranks second in terms of microbial communities, following the gut (Deo and Deshmukh, 2019). More critically, previous research has shown that salivary glands can secrete approximately 1 liter of saliva per day, which contains around 10^11^ bacteria (Shaalan et al., 2022). Accordingly, saliva samples exhibit more advantages in forensic investigations, including a high frequency of occurrence at crime scenes and rich sample content. Some studies have demonstrated that salivary microbial communities exhibited variations among different BMI populations (Bombin et al., 2022; de Andrade et al., 2020; Hashizume et al., 2015; Raju et al., 2019; Wells et al., 2022). For example, (Hashizume et al. 2015) observed an increase in the level of *mutans streptococci* in the saliva of morbidly obese patients after bariatric surgery. Additionally, (Raju et al. 2019) discovered a significant association between saliva microbiota diversity and BMI in Finnish children. However, none of these studies have specifically investigated the connection between BMI and salivary microbiota in forensic practices.

In this study, we collected saliva samples with different BMI categories and conducted 16S rRNA sequencing of these samples to address the following issues: (1) Assess the association between saliva microbial community and BMI in Chinese adults; (2) identify BMI-related microbial markers for forensic research; (3) develop a highly accurate prediction model for inferring BMI categories of saliva samples. This study aims to conduct a preliminary exploration of the intrinsic relationship between salivary microbiota and BMI, thereby deepening the understanding of their interaction patterns. By screening BMI-related biomarkers, we seek to identify a microbial signature that can reflect changes in individual BMI levels and to develop a preliminary model for inferring BMI. which is expected to provide a novel method for BMI inference in forensic science.

## 2 Materials and methods

### 2.1 Sample collection

As a pilot study, to minimize the influence of lifestyle, dietary habits, and other factors on the microbiological composition of saliva, we recruited subjects with similar lifestyles and dietary habits. For example, smoking, alcohol consumption, primary dining locations, dietary habits, including omnivorous or special diets, etc. Besides, the subjects shouldn't have any oral diseases (such as caries, periodontitis, xerostomia, or gingivitis) and a history of antibiotic injections or taken via oral in the past 3 months and no smoking history. After informing them of the purpose of this experiment and obtaining informed consent from all volunteers, a total of 76 volunteers were enrolled to collect saliva samples. All subjects were long-term residents of Guiyang, Guizhou, aged between 18 and 35 years, with the majority of them aged 20 to 22. Subjects were called to the laboratory between 4 and 6 p.m. Each individual was given a 15 mL sterile tube and asked to collect 2 mL of unstimulated saliva samples. The saliva was collected into pre-prepared EP tubes which were capped securely to prevent environmental contamination. The sealed tubes were then stored in the freezer for cryopreservation before DNA extraction. The detailed sample information was given in Supplementary Table 1. This study followed the guidelines of the ethics commission of Guizhou Medical University and was approved by the ethics commission of Guizhou Medical University (the approval number: 2023–212).

### 2.2 DNA extraction and 16S rRNA amplicon sequencing

In this study, we used the MagAtrract PowerSoil Pro DNA kit (Qiagen, Hilden, Germany) to extract microbial DNA in saliva and mocked samples. Using the ABI GeneAmp^®^ 9,700 PCR thermocycler (ABI, CA, USA), these DNA samples were utilized to perform PCR to amplify the 16S rRNA gene V3–V4 region using two primers as reported in (Liu et al. 2016). The reaction condition was listed as below: 95°C for 3 min; 27 cycles of 95°C for 30 s, 55°C for 30 s, 72°C for 45 s; 72°C for 10 min. According to the manufacturer's guidelines, the PCR product of these samples was obtained by electrophoresis with the 2% agarose gel, and the AxyPrep DNA Gel Extraction Kit (Axygen Biosciences, Union City, CA, USA) was used to purify these amplified products. Finally, a QuantusTM Fluorometer was utilized to quantify these purified products. On the Illumina NovaSeq 6,000 platform (Illumina, San Diego, USA), paired-end sequencing was performed on a pool of purified amplicons in equimolar quantities.

### 2.3 Bioinformatics analysis

Using an internal perl script, we firstly demultiplexed raw FASTQ files by fastp version 0.19.6 (Chen et al., 2018) and FLASH version 1.2.7 (Magoč and Salzberg, 2011). Meanwhile, the raw sequencing data have been deposited in the NCBI Sequence Read Archive (SRA) public database under accession number PRJNA1426909. Next, we removed chimera of these samples. Then these non-chimeric sequences were classified into operational taxonomic units (OTUs) with the criterion of a 97% sequence similarity level by the UPARSE 7.1 software (Edgar, 2013). Microbial annotation information of each sample at different taxonomy levels was obtained by the RDP classifier (Maidak et al., 1999) in comparison to the SILVA v138 database. The microbial data of each sample was standardized using a total sum scaling method. Next, the alpha diversity of these samples was assessed using mothur software (Schloss et al., 2009) version 1.30.2 and visualized by R software based on the normalized data. The beta diversity of these samples was assessed using the vegan version 2.6–4 and mixOmics version 6.1.3 packages of R software. The linear discriminant analysis effect size (LEfSe) approach (Segata et al., 2011) was used to identify species that displayed statistically significant differences between BMI groups by using the one-against-all algorithm. Moreover, the threshold of LDA was set to 2. Based on the cohort, we randomly classified these samples into training and testing sets at the ratio of 7:3. Finally, the microeco version 0.19.5 and random forest version 4.7–1.1 were used to develop the prediction model by the training set, and then the performance of the model was evaluated in testing samples. Finally, we also assessed the performance of the model to predict BMI in mocked evidentiary samples.

## 3 Results

### 3.1 Saliva microbiome profiling by 16S rRNA sequencing

In this study, we collected saliva samples from 76 healthy individuals to assess the microbial community of these samples by the 16S rRNA gene sequencing. After eliminating low-quality sequences and chimeras, we obtained a total of 5658950 optimized sequences. The length of optimized sequences ranged from 161 to 530 bp, with an average length of 423 bp (Supplementary Table 2). To classify the OTUs, we performed a 97% similarity clustering analysis and removed OTUs that matched chloroplast and mitochondrial sequences. This resulted in the identification of 1997 OTUs. Next, we conducted species annotation for the valid sequences at various taxonomic levels. The 1997 OTUs could be annotated to 27 phyla, 63 classes, 150 orders, 262 families, 534 genera, and 975 species (Supplementary Table 3). Based on the World Health Organization (WHO) Asian Chinese Reference Standard for BMI values (Consultation, 2004), the samples were divided into three groups: underweight (BMI < 18.5 kg/ m^2^), normal weight (18.5 kg/ m^2^ ≤ BMI < 24.0 kg/m^2^) and overweight (BMI ≥ 24.0 kg/m^2^).

### 3.2 Alpha diversity of salivary microbiota differs across BMI groups

**Figure 1:**
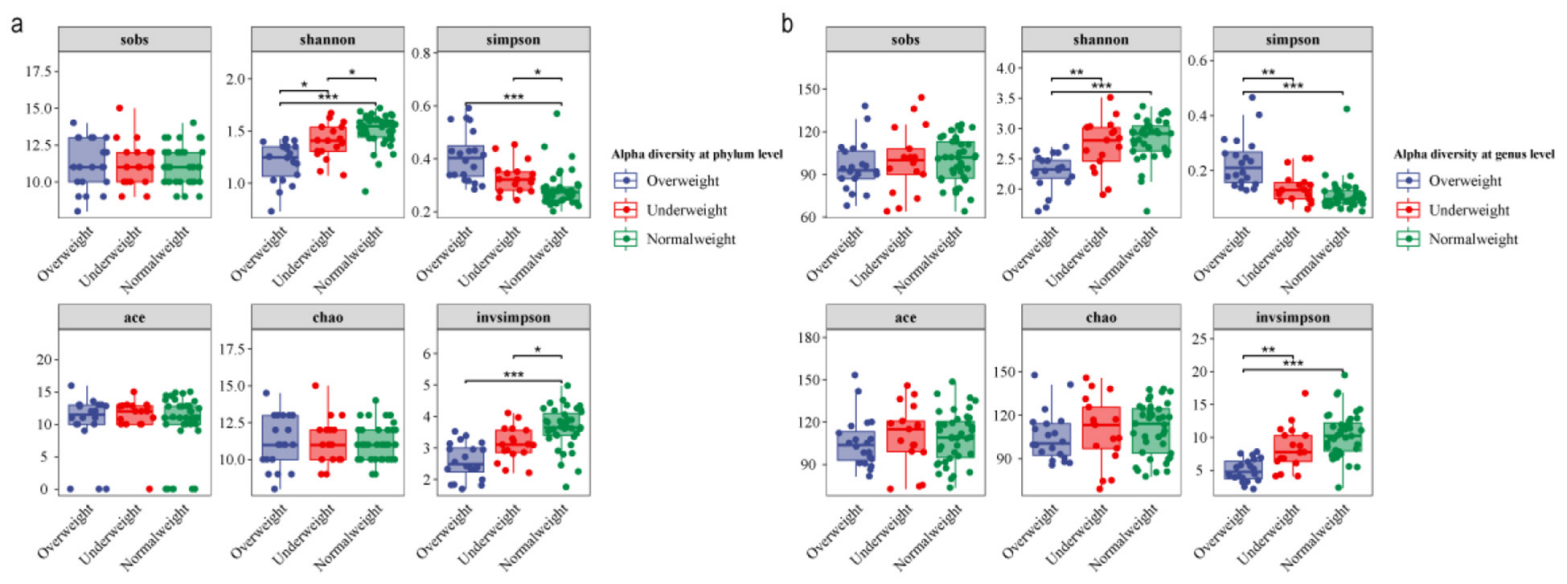
Alpha diversity of the 76 saliva samples at the phylum **(a)** and genus **(b)** levels. *P*-values between groups were obtained by the Kruskal-Wallis nonparametric test. Different colors represent different BMI groups. *p < 0.05, **p < 0.01, ***p < 0.001.

Because of the complexity of the salivary microbial communities, we utilized the alpha diversity index for visualization of microbial community richness in these samples. We found that the Shannon, and Simpson indices showed statistically significant differences between different BMI groups at the phylum and genus levels (*p*-value < 0.05) (Figure 1).

Additionally, we generated rarefaction curves of the Shannon index for all samples. The results indicated that as the amount of sample data increased, the rarefaction curves gradually stabilized, suggesting that the microbial profiles of the majority of samples were adequately represented (Supplementary Figure 1). We also investigated the potential influence of gender on the microbial communities by dividing the samples into male and female groups. Obtained results revealed that there were not statistically significant differences between genders for the alpha diversity at the phylum and genus level *(p*-value > 0.05) (Supplementary Figure 2).

### 3.3 BMI-dependent variations in salivary microbiota composition

**Figure 2:**
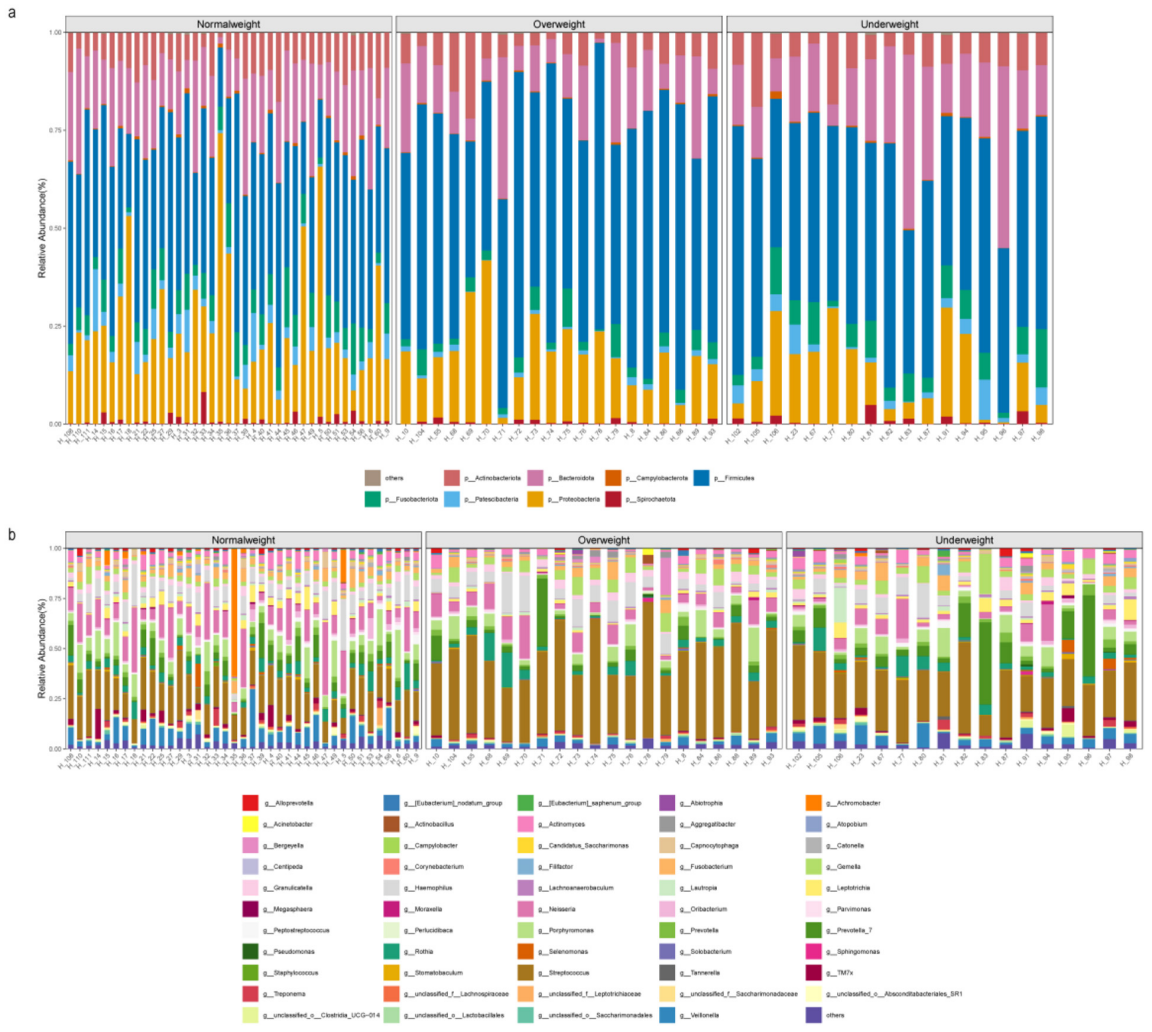
Relative abundance of microbial composition for 76 saliva samples at phylum **(a)** and genus **(b)** levels. Different colors represent different microbial profiles. Microbes with relative abundance below 1% were classified as others.

To gain insights into the compositional characteristics of microorganisms in the different groups, we analyzed the relative abundance of microbial community in these samples at the phylum and genus levels (Figure 2).

Our findings showed that *Firmicutes, Proteobacteria*, and *Bacteroidota* were the three most abundant phylum across all samples (Figure 2a). Furthermore, we observed that *Bacteroidota* had a higher relative abundance in the Underweight group compared to the other groups; *Firmicutes* had the lowest relative abundance in the normal weight group (Supplementary Figure 3a). At the genus level, we identified a total of 534 genera across all samples, with species exhibiting relative abundances below 1% classified as others. Most of these genera were present in all samples, indicating their relative stability among individuals. We found that Streptococcus and Gemella had the lowest abundances in the normal weight group compared to other groups; *Veillonella* and *TM7x* had the lowest abundances in the overweight group compared to other groups. Furthermore, *Prevotella_7, Leptotrichia*, and *Lautropia* showed the highest abundances in the underweight group and least abundant in the overweight group (Supplementary Figure 3b). Besides, we also observed inter-individual variations among samples from the same group. For example, sample H-35 displayed the high abundance of *Achromobacter* (Figure 2b).

### 3.4 BMI groups display significantly different salivary microbial community structures

**Figure 3:**
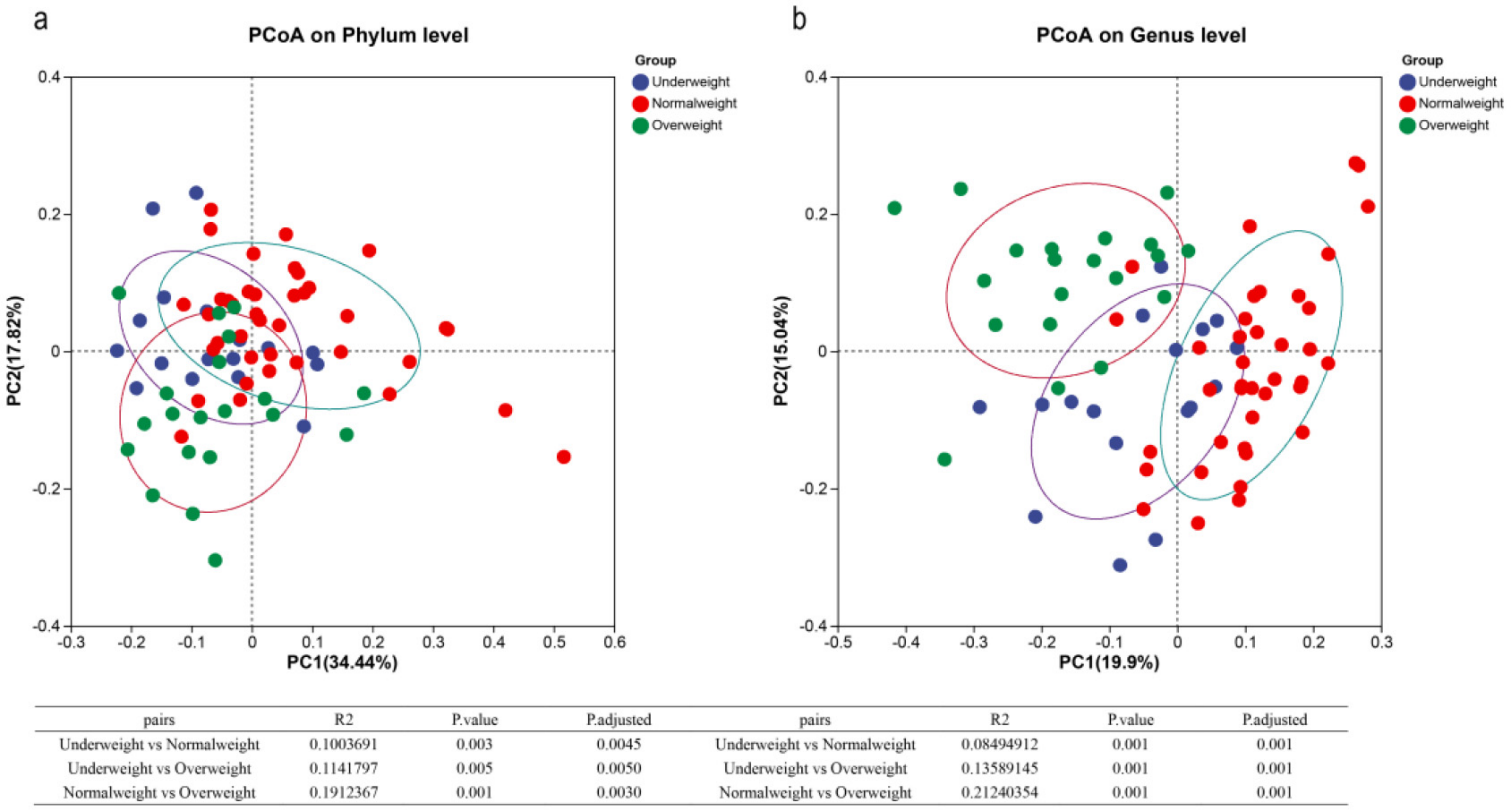
Principal co-ordinates analysis (PCoA) of 76 saliva samples with different BMI groups based on the Bray-Curtis distance at the phylum **(a)** and genus levels **(b)**.

To investigate variations in salivary microbiota across BMI groups, we performed PCoA analysis at the OTU, phylum, and genus levels. Significant differences in microbial composition were observed between BMI groups at all taxonomic levels. However, the *R*^2^ value at the OTU level was lower than that at the phylum and genus levels. The most pronounced between-group differences in salivary microbiota were observed at the genus level (Figure 3, Supplementary Figure 4).

At the phylum level, most samples from the overweight group clustered on the left, while most samples from the normal weight group clustered on the right. However, samples from the underweight group were scattered among the overweight and normal weight groups, which were difficult to differentiate from other groups. Similar phenomena could be observed at the genus level. More critically, most samples from the underweight group could also be differentiated from other groups at the genus level. We also conducted the PERMANOVA test to evaluate the differences of these saliva samples among different BMI groups based on the Bray-Curtis distance. The obtained results showed that the bacterial community structure was significantly different among these clusters grouped by the BMI at the OTU, phylum, and genus levels (*P-*adjusted < 0.01). These findings from the PCoA indicated that these BMI groups displayed significantly different distributions of the microbial community.

### 3.5 Identification of BMI-associated microbial markers

**Figure 4:**
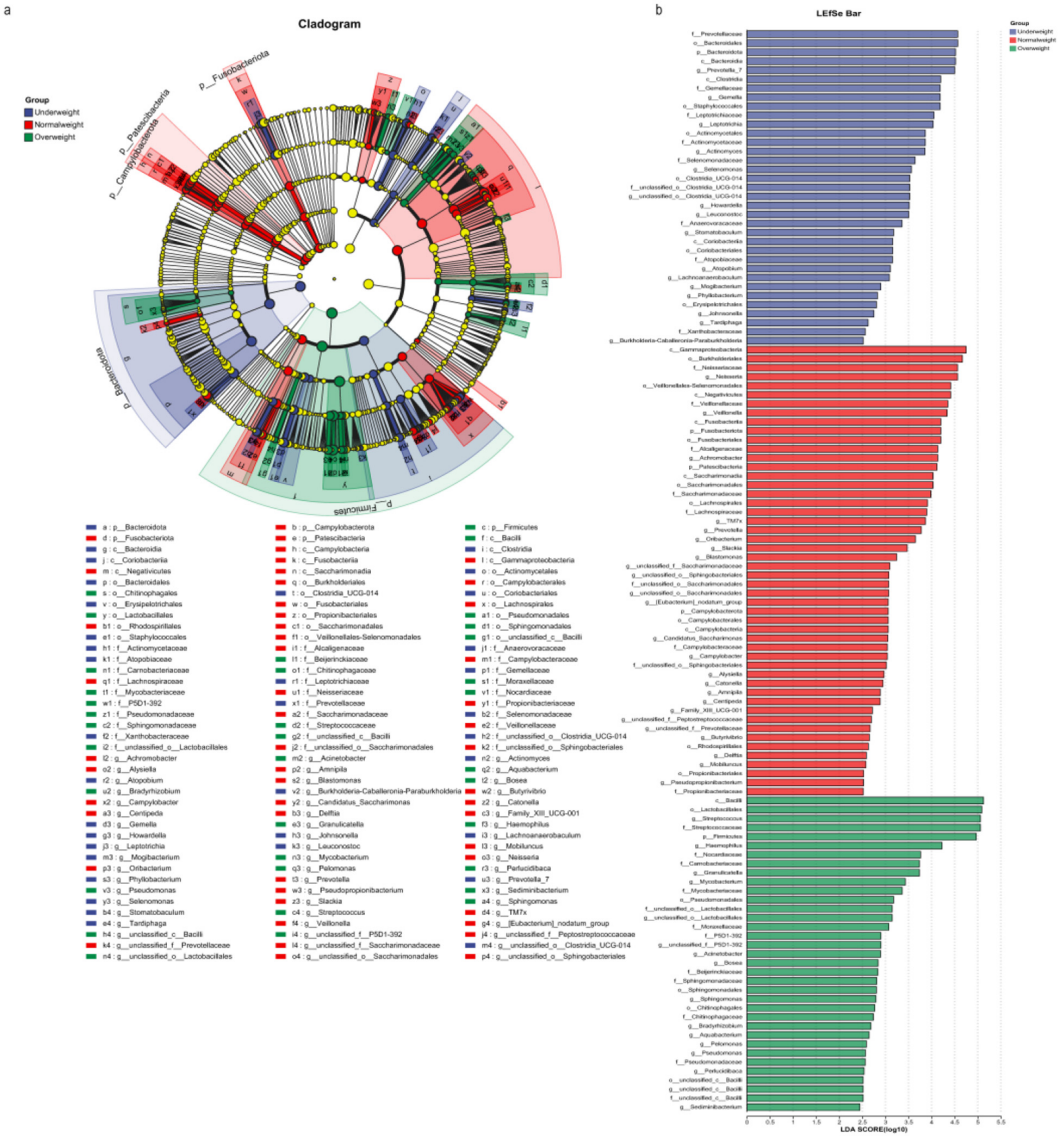
The results of linear discriminant analysis effect size (LEfSe) between different BMI groups. **(a)** the cladogram of taxa that showed significant differences between different BMI groups. **(b)** the bar graph of LDA scores of microbial profile that showed statistical differences among three groups (LDA >2).

Using LEfSe, we identified microbial markers that showed significant abundance differences between BMI groups, as illustrated in Figure 4a.

**Figure 5:**
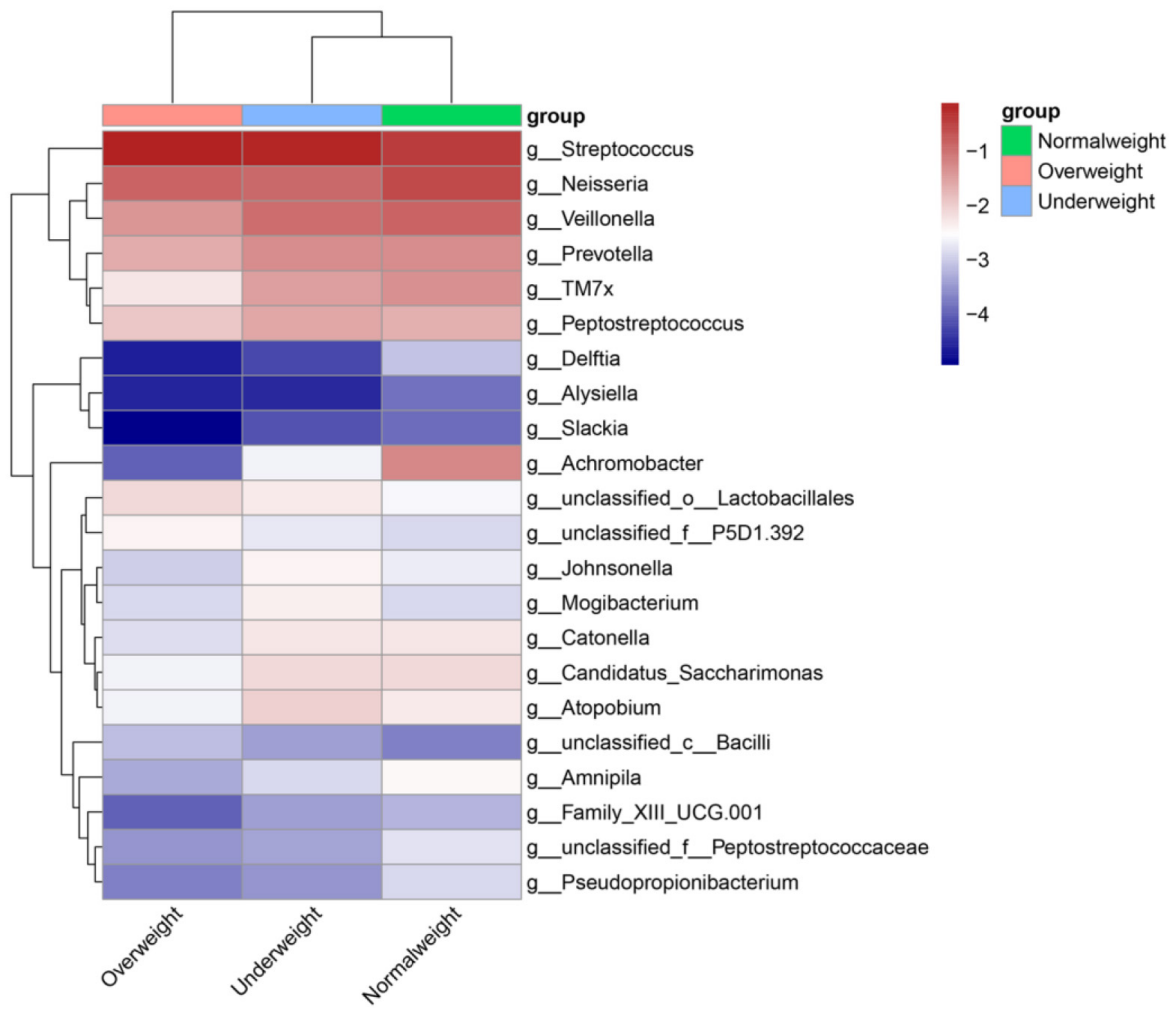
The heatmap of the relative abundance of 22 microbial markers in different BMI groups.

At the phylum level, five microbial markers exhibited significant differences among different BMI groups. Moving from the order to the genus level, more microbial markers that showed significant differences among different BMI groups could be observed. Furthermore, we conducted linear discriminant analysis (LDA) to evaluate the effect of species abundance on the different BMI groups. The results revealed that 120 microorganisms displayed significant abundance differences among different groups (Figure 4b, Supplementary Table 4). These microbial markers have the potential to serve as indicators for distinguishing different BMI groups. The results obtained from LEfSe analysis highlighted the significant between-group abundance difference. Notably, the results of PCoA showed that the discrimination between BMI groups was more significant at the genus level. Therefore, we mainly observed different species at the genus level. To further identify microbial markers highly related to BMI at the genus level, we utilized the Kruskal-Wallis rank sum test to calculate each microbe's importance for BMI inferences based on the microeco package with P-adjusted < 0.05, which identified 39 microbial markers highly correlated with BMI (Supplementary Figure 5, Supplementary Table 5). Finally, by cross-referencing the microbial markers calculated using the Kruskal-Wallis rank-sum test with those calculated at the genus level using the LDA algorithm (Figure 4, Supplementary Table 4), we identified 22 microbial markers highly correlated with BMI. The relative abundance of these 22 microbial markers in different BMI groups was further shown in Figure 5. We found that these species showed significant abundance differences among different BMI groups, e.g., *Achromobacter* and *Neisseria* had the highest abundance in the normal weight group. Meanwhile, we calculated the feature importance rankings for these 22 microbial markers (Supplementary Figure 6, Supplementary Table 6).

### 3.6 Random forest model validates 22 microbial markers for BMI classification

**Figure 6:**
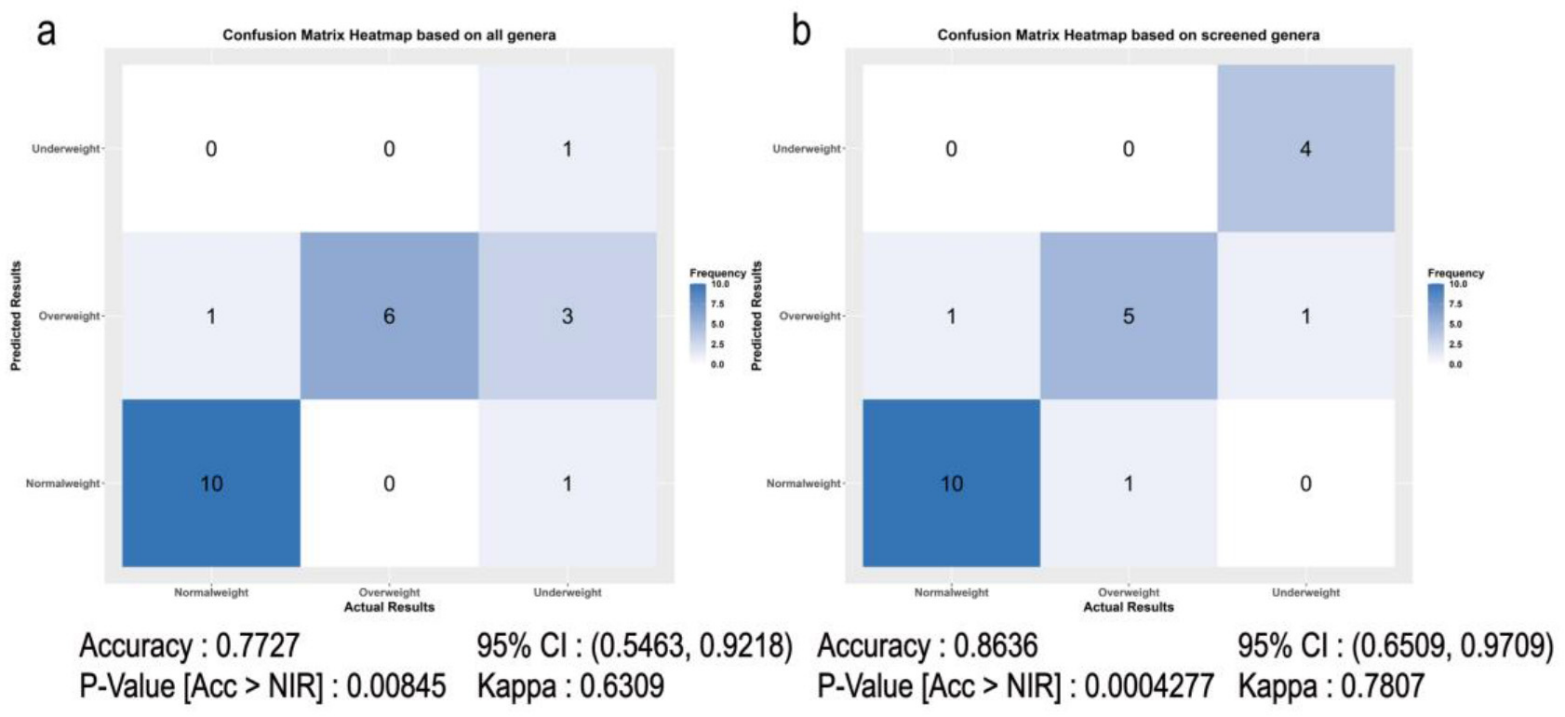
The predicted results of testing samples by the random forest model at the genus level. **(a)** the confusion matrix for predicted and actual results based on all genera. **(b)** the confusion matrix for predicted and actual results based on 22 microbial markers.

To validate the efficacy of the 22 microbial markers in discriminating different BMI groups, we constructed the random forest model. Firstly, 76 individuals were randomly classified into training and testing sets at the ratio of 7:3. Next, all genera and selected 22 microbial markers were used to build models based on training samples. The performance of constructed models was further evaluated in testing samples. The confusion matrix of predicted results and actual results in testing samples was shown in Figure 6.

We found that 19 samples were correctly classified into their BMI groups using the 22 selected microbial marker models, whereas only 17 samples were correctly classified using all genus-level models. In addition, the accuracy, Kappa coefficient, and *p*-value of the model built by 22 microbial markers were 86.36%, 0.7807, and 0.0004, respectively, which was also better than the results obtained by the model built by all genera. This suggested that these 22 microbial markers could be viewed as valuable markers for BMI prediction. Subsequently, we further evaluated the performance of the model in simulated samples from forensic crime scenes, as shown in Supplementary Figure 7. Unfortunately, the model manifested relatively poor performance in these simulated samples, and only 10 out of 30 samples were correctly classified into the corresponding groups.

## 4 Discussion

BMI acts as an important indicator of obesity in the human body and has broad applications in various fields. In forensic investigations, knowledge of a suspect's BMI can assist in targeting the suspect and narrowing down the investigation scope. This is particularly significant in cases where eyewitness testimony is the primary evidence, as the accuracy of such testimony is vital. Therefore, it is crucial to assess the scientific validity of eyewitness testimony based on biological information. While previous research has explored the associations between BMI and microorganisms in stool samples (Chen et al., 2021; Del Chierico et al., 2018; Stanislawski et al., 2019), the limited availability of stool samples hinders their routine application in forensic cases. Considering this limitation, we focused on saliva samples as they were more prevalent and accessible samples in forensic practice (Anzai-Kanto et al., 2005). As a preliminary exploration study, we aimed to explore the feasibility of identifying BMI-related markers in saliva microbiomes and to develop a preliminary model for inferring BMI. Our results demonstrate the potential of this approach. We also plan to apply this method to real-world cases to evaluate its practical potential in shortening investigation time and improving the accuracy of BMI identification.

In the study, we identified the most common bacteria at the phylum level, namely *Firmicutes, Proteobacteria, Bacteroidota, Actinobacteriota*, and *Fusobacteriota*. However, according to the LEfSe analyses, we only found that *Firmicutes, Bacteroidota*, and *Fusobacteriota* were defined as differentiating microorganisms among different BMI groups. Interestingly, LEfSe analysis identified Campylobacterota and Patescibacteria as differentially abundant in the normal weight group, although these phyla are not commonly prevalent in saliva. Meanwhile, a previous study found that the abundance of *Firmicutes, Bacteroidota, Fusobacteriota, Campylobacterota*, and *Patescibacteria* was significantly different among different BMI groups (Alqaderi et al., 2021). This suggests that even uncommon microbial species can still be considered as potential microbial markers for distinguishing different BMIs. However, caution is warranted in interpreting these taxa as direct BMI indicators. Patescibacteria, as symbionts dependent on their hosts, are commonly found in soil, freshwater, and the human microbiome, their presence in saliva may suggest exogenous ingestion. Similarly, certain members of the Campylobacterota have been implicated in gastrointestinal diseases such as diarrhea, mediating the onset and progression of these conditions and consequently leading to alterations in host BMI. We need to extend the scope of investigations and increase the depth of studies to further understand their interactions. At the genus level, *Neisseria, Achromobacter*, and *Veillonella* were defined as significantly differentiated microorganisms for the normal weight group in comparison to other groups. Furthermore, we also found that *Prevotella* exhibited relatively higher abundance in the normal weight group than other groups. Interestingly, a previous study also showed that the relative abundance of *Prevotella* had a positive correlation with BMI (Squillario et al., 2023), indicating that *Prevotella* might be a potential microbial marker for predicting BMI. We also found that *Streptococcus* exhibited relatively higher abundance in the overweight group than other groups, which was consistent with previous findings (Oduaran et al., 2020; Galle et al., 2020). Moreover, the significant variability of abundance for *Achromobacter* was also observed primarily in samples H-35 and H-5, indicating that *Achromobacter* showed relatively high individual variations among these samples, which might be related to their individual metabolic states and environmental conditions.

Further investigation is needed to explore the effect of these factors on the discrepancy of microbial community in different BMI groups to gain a better insight into the relationships between microbiome and BMI.

In analyzing these saliva samples, we observed that the normal weight and overweight groups could be clearly separated from each other by PCoA, while samples from the underweight group overlapped with samples from the normal weight and overweight groups. Several possible reasons could explain this phenomenon. Firstly, obesity can impact the body's metabolic rate, including glucose and fat metabolism, which may consequently lead to changes in the composition of the salivary microbial community. Therefore, it's easy to distinguish the overweight group from other groups. Secondly, differences in human adaptation to environmental factors could also play a role in shaping the variations observed in microbial communities. To enhance the ability of salivary microbial communities to accurately recognize different BMI categories, it is crucial to evaluate the effects of individual physical status, environmental factors, and other potential confounding variables. Additionally, more samples, especially for samples with underweight and overweight, should be collected to better explore the association between salivary microbial communities and BMI.

Even though we identified 22 potential microbial profiles that could be viewed as beneficial markers for inferring BMI of saliva samples, there were still some shortcomings remaining to be overcome. Firstly, some confounding factors (like age, lifestyle, dietary habits, and individual metabolic states) that may influence microbial communities were not considered in this study when screening BMI-related markers, which may reduce the application values of these microbial markers in forensic practice, especially for those unknown saliva samples. Besides, saliva-related samples rather than saliva are the common biological samples found in forensic scenes. These saliva-related samples are usually exposed to various conditions that also exert significant influences on the microbial community. Therefore, in the following study, we need to collect more saliva-related samples found in crime scenes to further evaluate the practical application efficiency of these microbial markers to infer BMI categories and to investigate the effect of *in vitro* exposure factors on the BMI-related microbial markers. This additional research can help us better comprehend the microbial dynamics associated with BMI and improve the accuracy of BMI inference in forensic practice.

## 5 Conclusion

In conclusion, we found that *Streptococcus, Veillonella, TM7x, Prevotella_7, Leptotrichia*, and *Lautropia* genera exhibited different abundance distributions among different BMI groups. In addition, 22 microbial markers were identified as the BMI-related markers by the LDA algorithm and the Kruskal-Wallis rank sum test, which could be employed as the promising indicators to infer the BMI of saliva samples. However, the efficacy of the screened microbial markers for predicting BMI was affected by some factors, such as lifestyle, dietary habits and saliva status. So, before using this method in forensic practice, further in-depth studies are needed to investigate the impact of oral status, environment, lifestyle, individual metabolic states, inter-individual variability, and other factors on inferring BMI.

## Data Availability

The raw data from this study have been deposited in the NCBI Sequence Read Archive (SRA) public database. This data is available in NCBI under BioProject PRJNA1426909. (http://www.ncbi.nlm.nih.gov/bioproject/1426909).
