# Supplementary material for: Microbial community variations in human salivary samples with different body mass index for forensic research: a pilot study
Source: Front Microbiol. 2026 Apr 21;17:1783246. doi: 10.3389/fmicb.2026.1783246 (PMC13139067; doi:10.3389/fmicb.2026.1783246)
Supplement: Supplementary file 1 [file Table_1.docx]

**Supplementary Figures**


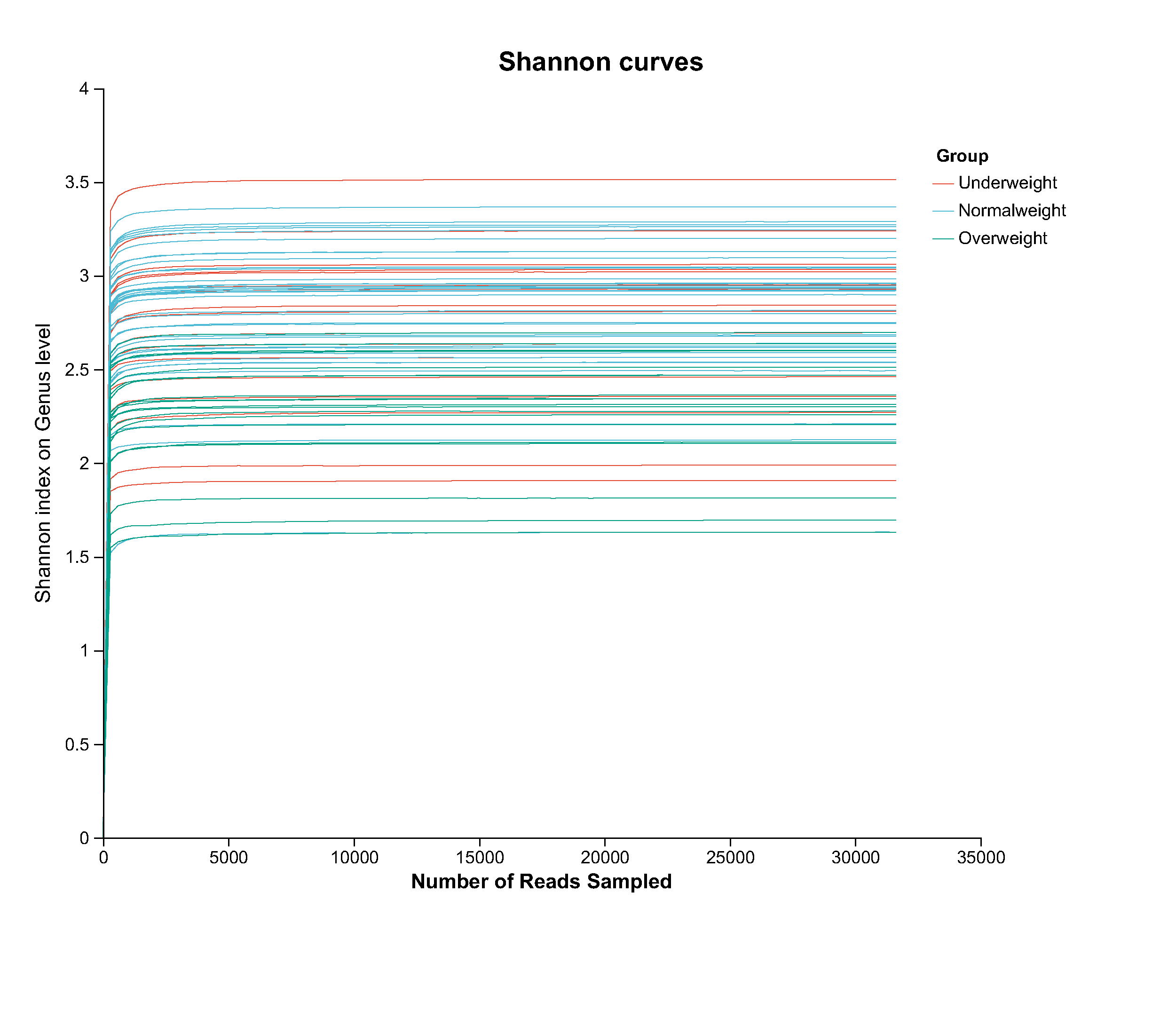


**Figure S1:** Rarefaction curves of the Shannon index at the genus level. Different colors represent different BMI groups.

**
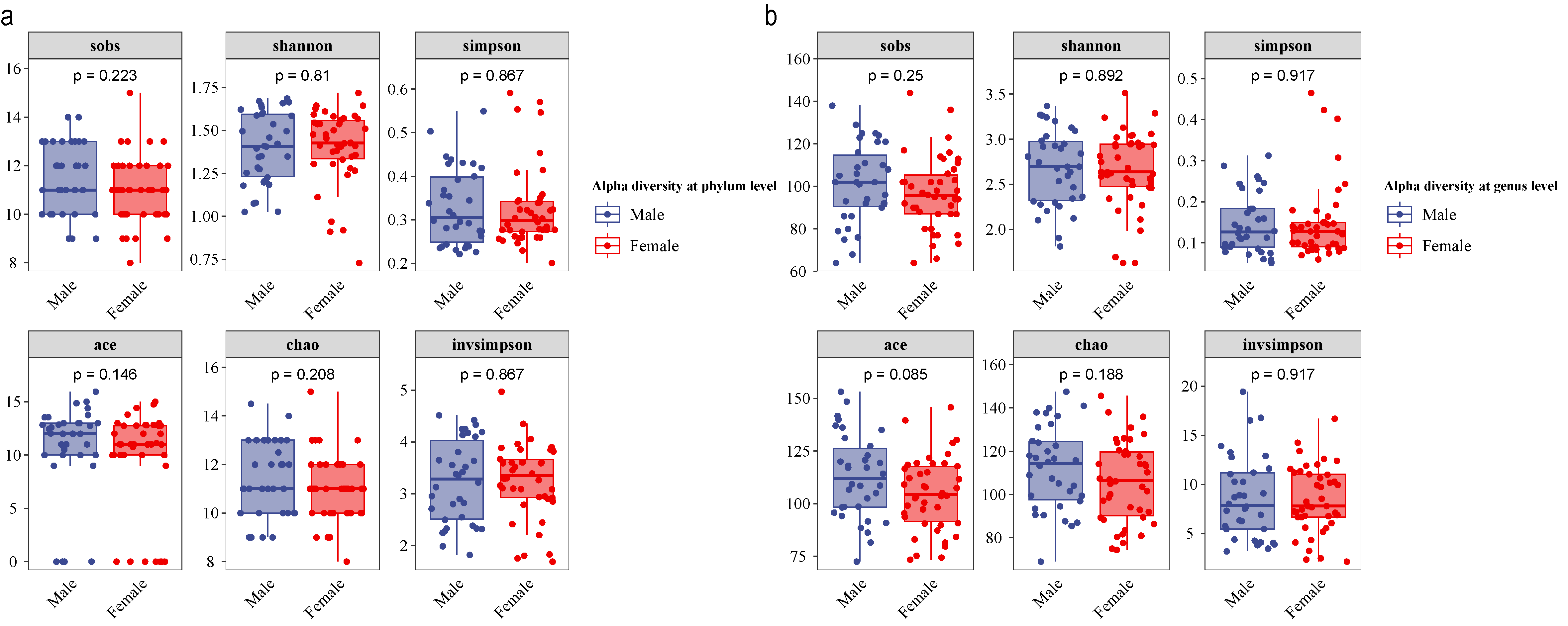
**

**Figure S2:** Alpha diversity of the 76 saliva samples at the phylum (a) and genus (b) levels. *P*-values between males and females were obtained by the Kruskal-Wallis nonparametric test.

**
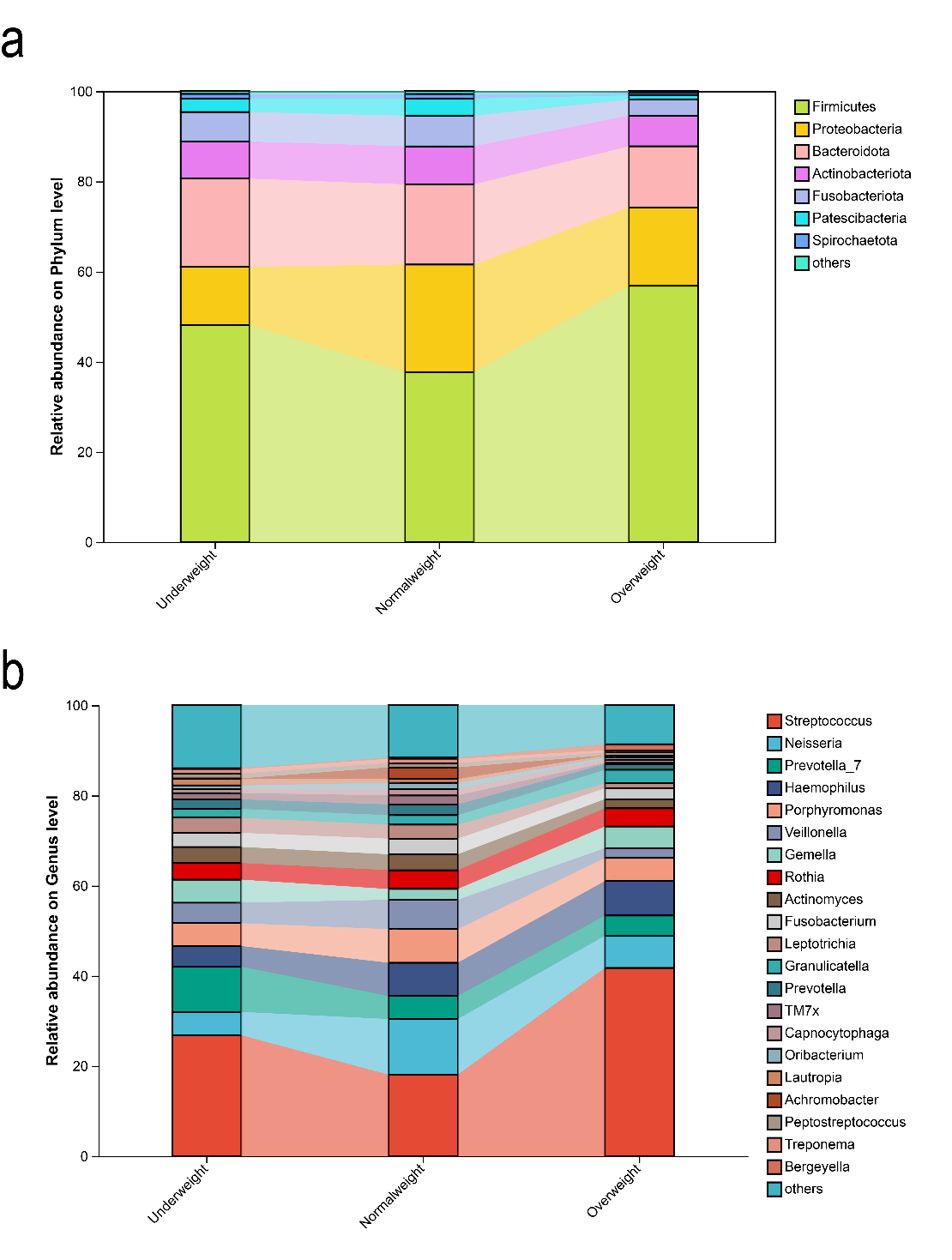
**

Figure S3: Stacked bar charts showing the relative abundances of microbial communities in saliva samples from different groups at the phylum (a) and genus (b) levels. Different colors represent different microbial profiles. Microbes with relative abundance below 1% were classified as others.

**
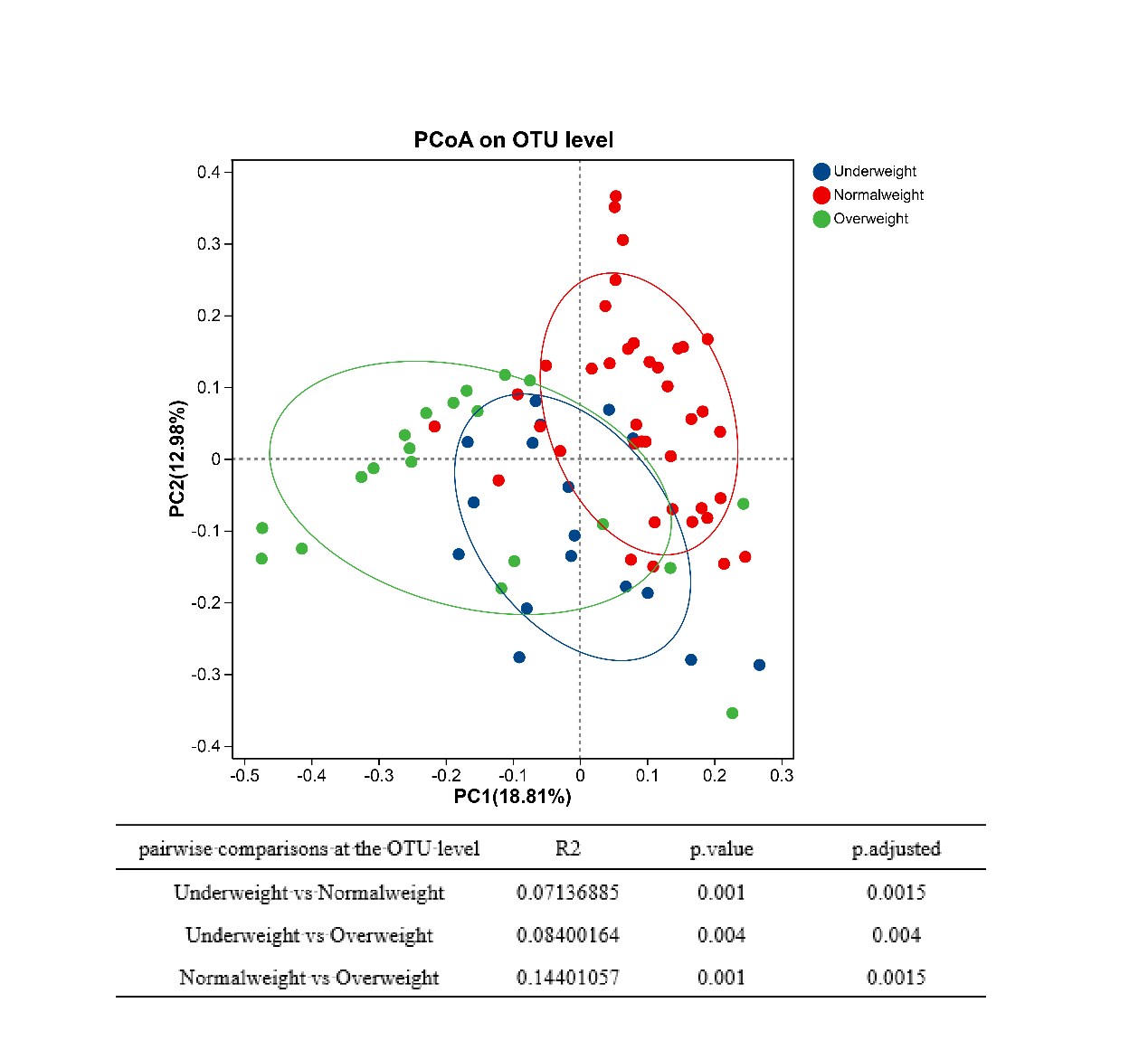
**

**Figure S4:** Principal co-ordinates analysis (PCoA) of 76 saliva samples with different BMI groups based on the Bray-Curtis distance at the OTU levels

**
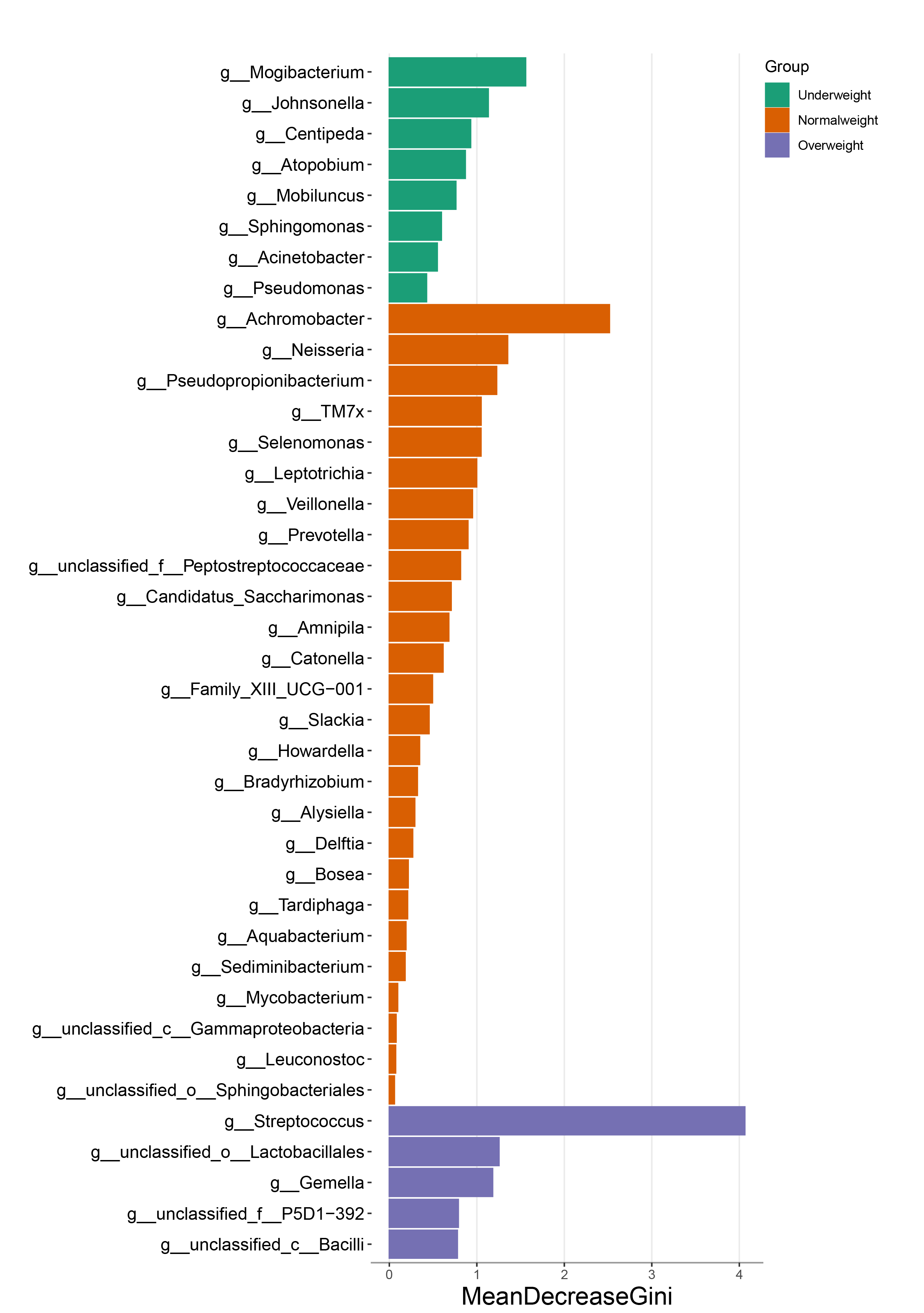
**

**Figure S5:** The mean decrease gini of selected microbial markers among the three groups.


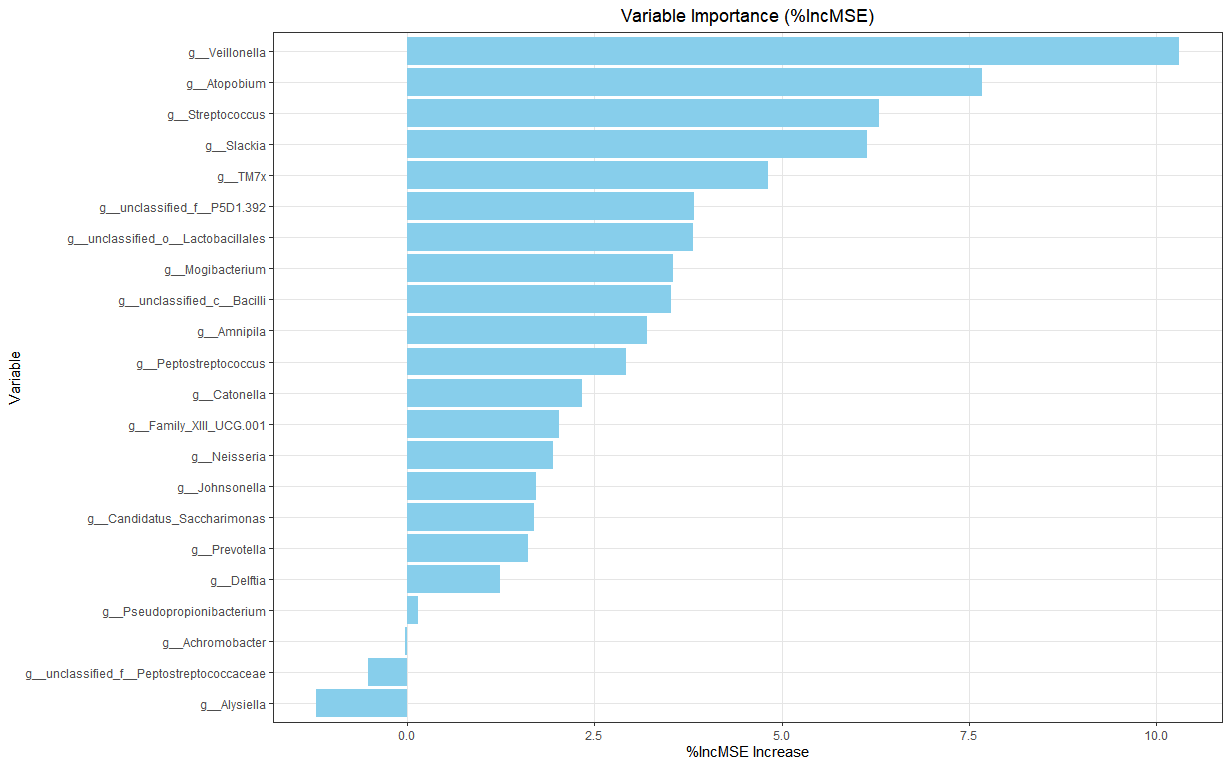


**Figure S6:** Ranking of Feature Importance for the 22 Selected Microbial Biomarkers.

**
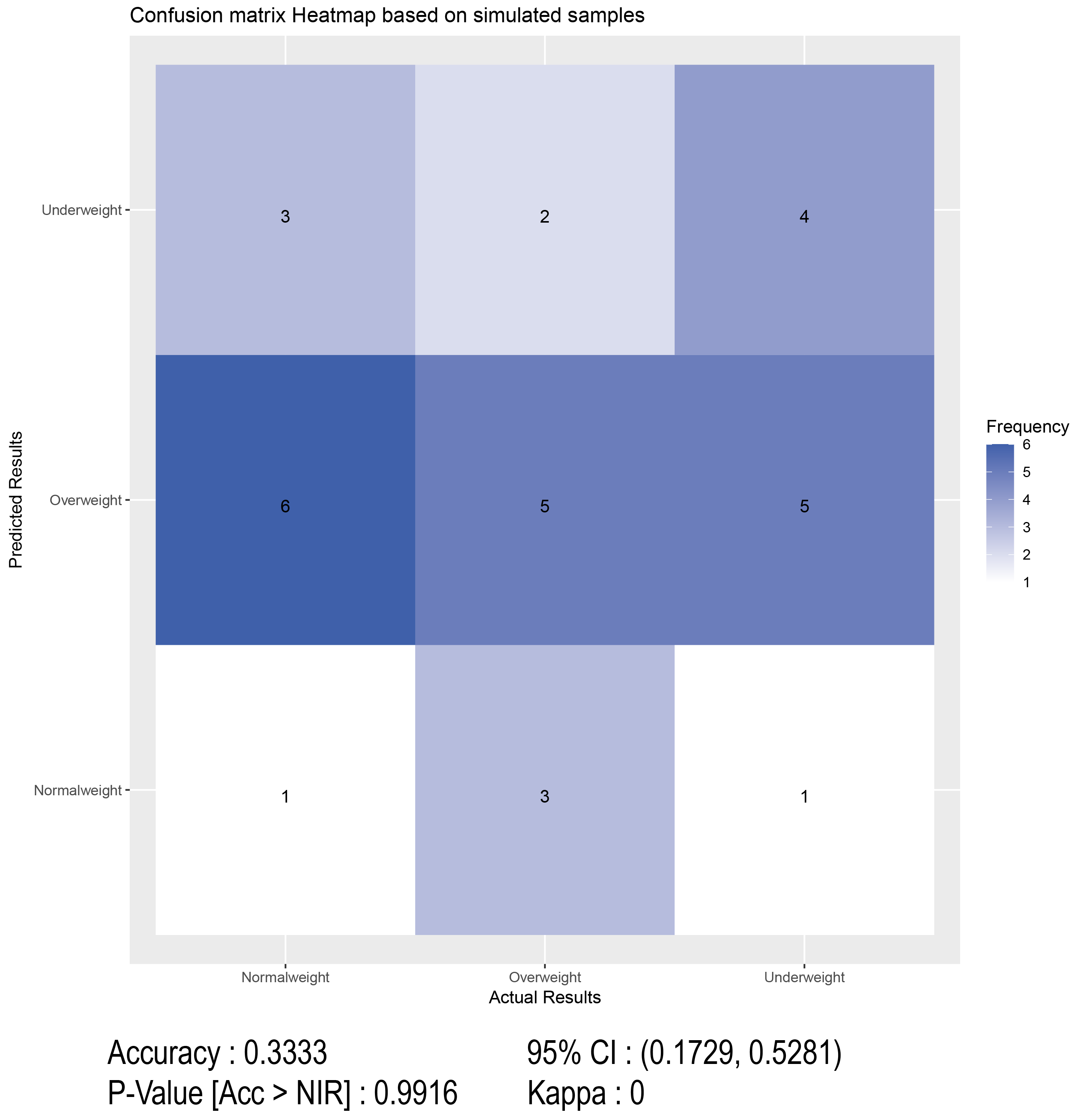
**

**Figure S7:** Classification prediction results of 30 simulated samples by the random forest model constructed based on screened microbial markers.
